# Comparative proteomic analysis of the brain and colon in three rat models of irritable bowel syndrome

**DOI:** 10.1186/s12953-020-0157-9

**Published:** 2020-02-24

**Authors:** Beihua Zhang, Hong Xue, Wei Wang, Ting Chen, Min Su, Nan Kang, Jianqin Yang, Zhaoxiang Bian, Fengyun Wang, Xudong Tang

**Affiliations:** 1 Department of Gastrointestinal Medicine, Xiyuan Hospital China Academy of Traditional Chinese Medical Sciences, Beijing, 100091 China; 2 Department of Gastrointestinal Medicine, Affiliated Hospital of Jining Medical University, Jining, Shandong China; 3 Department of Gastrointestinal Medicine, Wangjing Hospital, China Academy of Chinese Medical Sciences, Beijing, China; 4 Chinese Medicine Clinical Study Center, Jockey Club School of Chinese Medicine, Hong Kong Baptist University, Kowloon Tong, Hong Kong SAR China

**Keywords:** Irritable bowel syndrome models, Proteomics, Brain, Colon

## Abstract

**Background:**

Irritable bowel syndrome (IBS) has been gradually recognized as a disorder of the brain-gut interaction, but the molecular changes in the brain and colon that occur in disease development remain poorly understood. We employed proteomic analysis to identify differentially expressed proteins in both the brain and colon of three IBS models.

**Methods:**

To explore the relevant protein abundance changes in the brain and colon, isobaric tags for relative and absolute quantitation (iTRAQ), liquid chromatography and tandem mass spectrometry (LC-MS) and Western blotting methods were used in three IBS models, including maternal separation (MS, group B), chronic wrap restraint stress (CWRS, group C) and a combination of MS and CWRS (group D).

**Results:**

We identified 153, 280, and 239 proteins that were common and differentially expressed in the two tissue types of groups B, C and D, respectively; 43 differentially expressed proteins showed the same expression changes among the three groups, including 25 proteins upregulated in the colon and downregulated in the brain, 7 proteins downregulated in the colon and upregulated in the brain, and 3 proteins upregulated and 8 downregulated in both tissues. Gene ontology analysis showed that the differentially expressed proteins were mainly associated with cellular assembly and organization and cellular function and maintenance. Protein interaction network and Kyoto Encyclopedia of Genes and Genomes (KEGG) pathway analysis indicated that the differentiated proteins were mainly involved in the protein ubiquitination pathway and mitochondrial dysfunction.

**Conclusions:**

Taken together, the data presented represent a comprehensive and quantitative proteomic analysis of the brain and colon in IBS models, providing new evidence of an abnormal brain-gut interaction in IBS. These data may be useful for further investigation of potential targets in the diagnosis and treatment of IBS.

## Background

Irritable bowel syndrome (IBS) is one of the most common gastrointestinal diseases with high prevalence and is a chronic disease characterized by visceral pain and/or discomfort, visceral hypersensitivity and abnormal motor responses [1]. Although the etiopathogenesis of IBS is multifactorial and not completely elucidated, current concepts attribute an important role to a complex interplay between the gastrointestinal (GI) system and the central nervous system (CNS) [2–4]. IBS is commonly recognized as a brain-gut disorder, and psychosocial stress is its most widely acknowledged risk factor [5–7]. Through this brain-gut connection, we may explain why stress and psychological factors are linked so closely to gut function and dysfunction, as well as gastrointestinal symptoms. Thus, we must investigate the mechanism of the brain-gut interaction to understand the pathophysiology of IBS.

The brain-gut axis (BGA) and gut-brain axis (GBA) include the enteric nervous system (ENS), the gut wall in the periphery, the CNS, and the hypothalamic-pituitary-adrenal (HPA) axis [2, 8]. The bidirectional communication between the gut and the CNS is based on neural, endocrine and neuroimmune pathways. Both brain-gut and gut-brain dysfunctions may lead to gastrointestinal disorders such as IBS. Further investigation of the BGA has revealed that the ENS and CNS share many features, including certain biologically active peptides [9], and the molecular changes in the brain and colon that occur in disease development remain poorly understood.

Recently, proteomic analysis has become one of the best strategies to reveal protein structure and functional interactions among cellular or secreted proteins on a large scale [10, 11]. isobaric tags for relative and absolute quantitation (iTRAQ) has become one of the major quantification tools in differential proteomic research due to many advantages over older 2-D electrophoresis methods, including the reduction in analytical bias and the detection of low-abundance proteins [12].

Some previous studies have focused on detecting differentially expressed proteins in the urine of IBS patients [13, 14] or in the colons of animal models [14–16], but few studies have concentrated on differentially expressed proteins in the brain. In the present study, we aimed to construct a proteomics map for brain and colon tissue and to identify differentially expressed proteins between the IBS model and control groups. To achieve the objectives, we used iTRAQ combined with liquid chromatography and tandem mass spectrometry (LC-MS) for proteomic analyses in three IBS-D rat models.

## Methods

### Ethics statement

Fifteen pregnant Sprague-Dawley rats (pregnant time: 18 ± 2 d) were obtained from the Animal Centre of Xiyuan Hospital, China Academy of Chinese Medical Sciences, Beijing, China. The rats were housed in stainless-steel hanging cages in a colony room maintained under a 12-h light/dark cycle with a room temperature of 22 ± 1 °C and humidity of 65–70%. Water and food were available ad libitum. The experimental procedures followed the guidelines and practices of the Animal Care Ethics Committee of Beijing. The procedures were conducted in accordance with the Beijing Administration Office Committee of Laboratory Animals. The protocols performed in studies involving animals were in accordance with the Animal Care and Use Committee of Xiyuan Hospital, China Academy of Chinese Medical Sciences.

### The maternal separation (MS) model

According to relevant references [17, 18], from 09:00 to 12:00 on postnatal day (PND) 2, 70 pups were randomly removed from their maternity cages and placed into separate identical cages until the end of the manipulation. After 3 h of separation, the pups were returned to their maternity cages until weaning on PND 22. Normally handled (NH) pups remained undisturbed in their home cage with the dam. All pups were weaned on PND 22. Only male pups were used in the present study, and on PND 60, 10 male rats were randomly allocated to a series of wrap restraint stress sessions.

### The chronic wrap restrain stress (CWRS) model

The wrap restraint stress model is commonly applied as a model for human IBS [19].

The stress session was performed between 09:00 to 12:00 to minimize the influence of circadian rhythms. The rats’ upper forelimbs and thoracic trunk were wrapped using adhesive tape for 3 h daily over three consecutive weeks.

### Experimental animal grouping

**Fig. 1:**
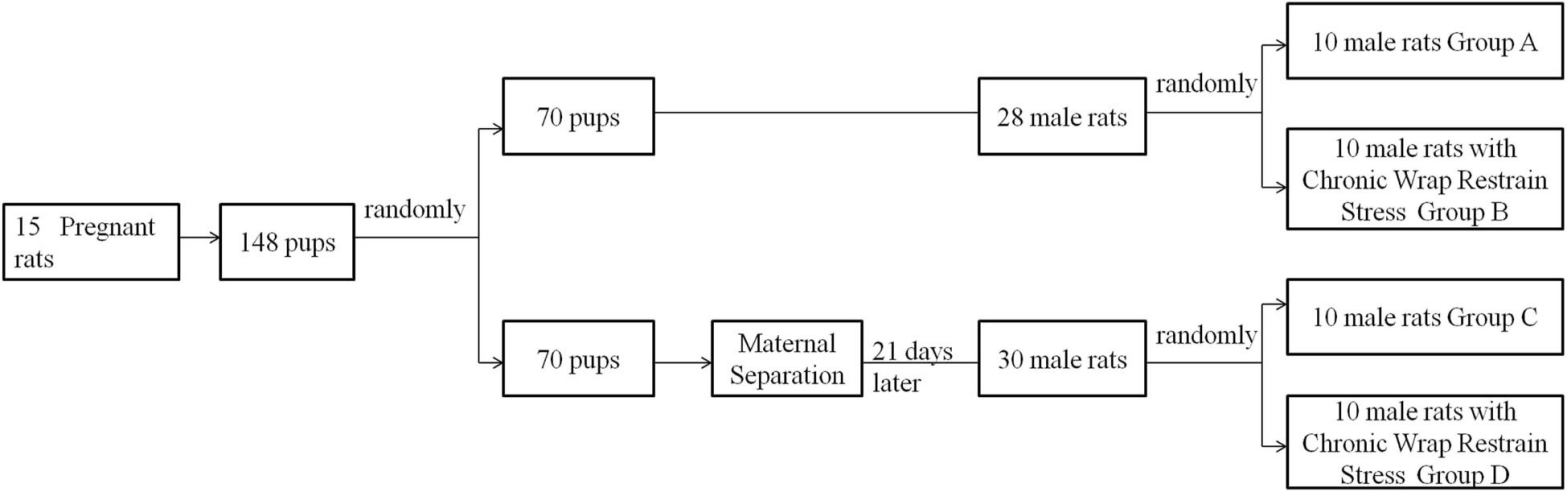
Flow chart of experimental animal grouping

Rats were randomly divided into 4 groups of 10 animals each, as shown in Fig. 1. Group A is the control, group B is the CWRS group, and group C is the MS group. Group D is a superposition of MS and CWRS interventions in which CWRS was performed on adult rats who were separated from the mother (underwent MS) as a pup. At the end of the studies on day 81, animals were euthanized by intraperitoneal injection of 7% chloral hydrate followed by cervical dislocation, and the colon and brain were collected for further investigation.

### Behavioral testing of the IBS models

The behavioral response of the IBS models was assessed by measuring the abdominal withdrawal reflex (AWR) using a semiquantitative scoring system [20]. The procedure was performed according to the previous reference. The assignment of an AWR score based on the behavioral scale was as follows: grade 0, no behavioral response; grade 1, brief head movement only at the onset of the stimulus; grade 2, contraction of abdominal muscles but no lifting of the abdomen off the platform; grade 3, strong contraction of the abdominal muscles with lifting of the abdomen; and grade 4, a severe contraction of the abdominal muscles leading to body arching and lifting of the pelvis.

### Protein extraction and digestion

Samples were ground into powder in liquid nitrogen, and total proteins were extracted using the cold acetone method. First, 10% trichloroacetic acid (TCA) in acetone was added to the samples, followed by incubation at − 20 °C for 2 h and centrifugation at 20,000×g at 4 °C for 30 min. The white pellet was collected, and the supernatant was discarded. The pellets were resuspended in cold acetone and centrifuged again at 20,000×g for 30 min at 4 °C. The precipitate was washed with chilled acetone three times. The pellet was resuspended in 1 ml of protein extraction reagent (8 M urea, 4% (w/v) CHAPS, 30 mM HEPES, 1 mM PMSF, 2 mM EDTA, and 10 mM DTT) and sonicated for 5 min. The samples were then centrifuged at 20,000×g for 30 min at 4 °C, the pellets were discarded, and the supernatant was used for liquid digestion. To reduce disulfide bonds in the proteins of the supernatant, 10 mM DTT (final concentration) was added and incubated at 56 °C for 1 h. Subsequently, 55 mM IAM (final concentration) was added to block the cysteines, and the solution was incubated for 1 h in the darkroom. The supernatant was mixed well with 4x volume of chilled acetone for 3 h at − 20 °C to precipitate proteins. After centrifugation at 4 °C and 30,000 g, the supernatant was discarded, and the pellet was dissolved in 300 μl 0.5 M tetraethylammonium bicarbonate (TEAB; Applied Biosystems, Milan, Italy) and sonicated at 180 W for 3 min. Finally, samples were centrifuged at 4 °C and 30,000 g for 15 min. The protein concentration was determined using the Bradford assay. A 100-μl aliquot of each protein sample was combined with an equal volume of TEAB, pH 8.5, followed by treatment with trypsin (3.3 μg trypsin/100 μg total protein).

### iTRAQ labeling and strong Cation-exchange (SCX) high-performance liquid chromatography (HPLC)

The peptides were labeled with 8-plex iTRAQ reagents (AB Sciex, Foster City, CA, USA) according to the manufacturer’s protocol. The samples were fractionated using an HPLC system (Shimadzu, Japan) equipped with an SCX column (Luna 5-μm column, 4.6 mm I.D. × 250 mm, 5 μm, 100 Å; Phenomenex, Torrance, CA). The retained peptides were eluted by a step linear elution program using buffer A (10 mM KH_2_PO4 in 25% ACN, pH 3.0) and buffer B (2 M KCl, 10 mM KH_2_PO_4_ in 25% ACN, pH 3.0), and the fractions were collected in 1.5-ml microfuge tubes. The flow rate was set at 1 mL/min. The following gradient was applied: for 50 min, 100% buffer A was used; from 50~51 min, the buffer B concentration was increased to 5%; from 51~66 min, the buffer B concentration was increased to 30%; from 66~76 min, the buffer B concentration was increased to 50% and then maintained for 10 min; and from 81 to 91 min, the buffer B concentration was increased to 100%. The peptide information of brain and colon for iTRAQ experiment were shown in Additional file 1.

### Peptide identification by Nano-RP HPLC and mass spectrometry

The desalting protocol via C18 reverse-phase chromatography was performed as described previously. The eluted fractions were delivered onto a nano-RP column (5-μm Hypersil C18, 75 μm × 100 mm, Thermo Fisher Scientific, Waltham, MA, USA) mounted in a Prominence Nano HPLC system (Shimadzu, Nakagyo-ku, Kyoto, Japan). The peptides were separated using a C18 analytical reverse-phase column at a solvent flow rate of 400 nL/min (solution A, 0.1% formic acid; solution B, 95% acetonitrile/0.1% formic acid) for 120 min. A linear LC gradient profile was used to elute the peptides from the column. After sample injection, the column was equilibrated with 5% solution B for 10 min, and the following gradient schedule was then initiated: 30% solution B at 40 min; 60% solution B at 45 min; 80% solution B at 48 min, which was maintained for 10 min; and 5% solution B at 58 min, which was held for 15 min before ramping back down to the initial solvent conditions. The fractions were analyzed using Q-Exactive (Thermo Fisher Scientific, Waltham, MA, USA), in the positive ion mode, with an m/z between 350 and 2000, full-scan resolution at 70,000, MS/MS scan resolution at 17,500 with a minimum signal threshold 1E+ 5 and isolation width at 2 m/z. Up to the top 20 most abundant isotope patterns with charge ≥2 and ≦7 from the survey scan were selected and fragmented by higher energy collisional dissociation (HCD) with normalized collision energies of 28%.

### Data analysis and functional analysis of differentially expressed proteins

In the present study, to ensure sufficient biological replicates and the reliability of the data, we collected colon tissue from five rats and brain tissue from four rats per group for proteomic analysis. The raw MS/MS data were converted into MGF format by Proteome Discoverer 1.3 (Thermo Fisher Scientific, Waltham, MA, USA). The UniProt database was downloaded and integrated into the Mascot search engine, version 2.3.01, through its database maintenance unit. Several parameters in Mascot were set for peptide searching: trypsin was specified as the digestion enzyme, cysteine carbamidomethylation as a fixed modification, iTRAQ 8Plex on the N-terminal residue, iTRAQ 8Plex on tyrosine (Y), iTRAQ 8Plex on lysine (K), glutamine as pyroglutamic acid, and oxidation on methionine (M) as a variable modification.

The Mascot search results were exported into a DAT FILE and quantified using Scaffold version 3.0 software. The fold changes in protein abundance were defined as the median ratio of all significantly matched spectra with tag signals. We performed a functional category gene enrichment test using Blast 2GO to determine whether the differentially expressed proteins were significantly enriched in any functional subcategories. The number of differentially expressed proteins was imported into IPA (Ingenuity Pathway Analysis) software and used to identify the protein biological pathway analysis based on the Gene Ontology (GO) and UniProt database.

### Western blot analysis

The proteins (20 μg) were separated by 8% SDS/PAGE and then electroblotted onto a PVDF membrane (Millipore), which was then washed for 10 min with TBST and immersed in blocking buffer containing 5% nonfat dry milk in TBST for 1 h at 25 °C. The blot was washed with TBST and then incubated with a rabbit polyclonal primary GAP43 antibody (Abcam, 1:5000 ab75810) overnight at 4 °C. After the blot was washed in TBST, it was incubated with a secondary antibody against rabbit IgG (Santa Cruz 1:2000) for 1 h at 25 °C. The blot was finally washed with TBST, and the protein bands were visualized with a chemiluminescence system (ECLPlus, Applygen Technologies, Inc.).

### Statistical analysis

Data were collected and analyzed using SAS8.0 software. Comparisons between two groups were made with Mann−Whitney U tests, and among multiple groups with 2-way ANOVA and Tukey’s post hoc test; an associated probability (*P* value) of less than 0.05 was considered significant.

## Results

### Identification of three IBS models

**Fig. 2:**
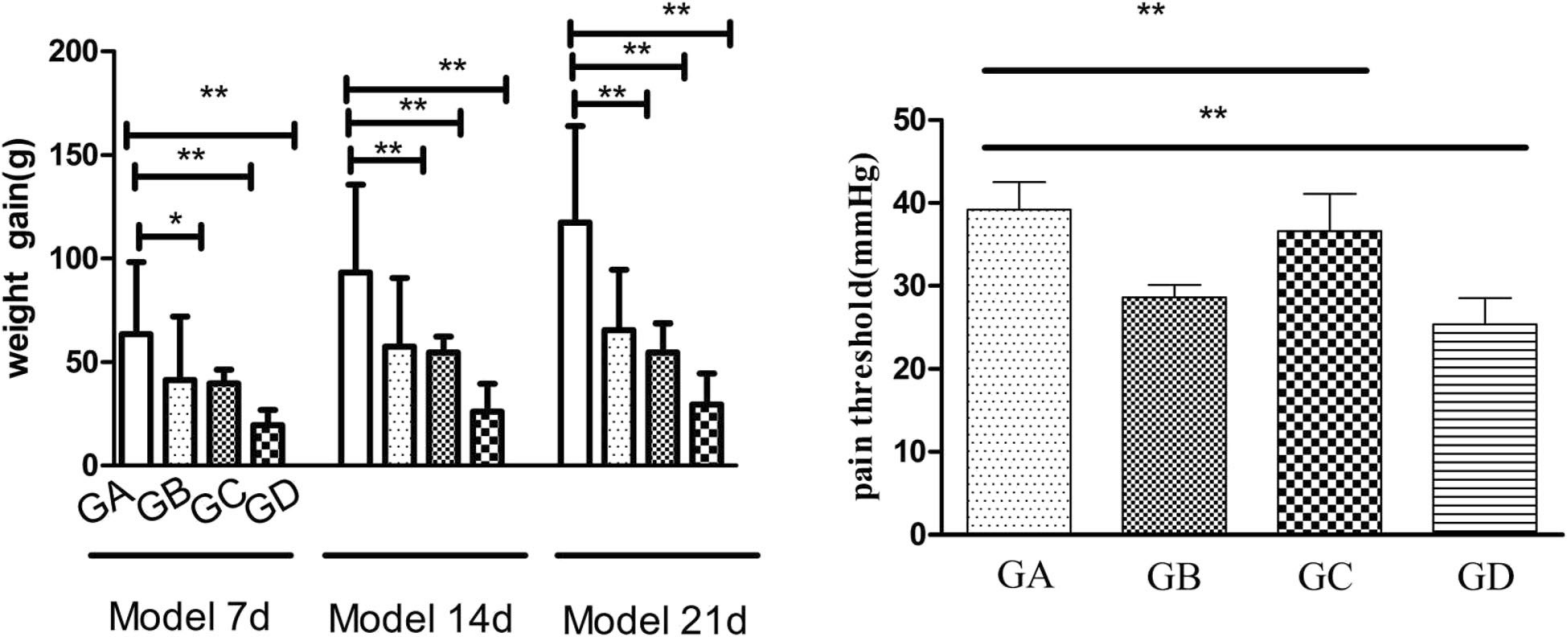
The pain threshold (right) and rat weight (left) of three IBS models compared to control rats. Data are expressed as the mean ± SEM. (*n* = 10). ^*^P < 0.05; ^**^P < 0.01. GA: group A, GB: group B, GC: group C

In the present study, three IBS models, including MS, CWRS, and a combination of maternal separation with chronic wrap restraint, were established. These models (MS and CWRS) are known to induce visceral hypersensitivity [21], which is one of the main signs of IBS. The establishment of the IBS models was verified by measurements of weight and visceral sensation. The rats of the three model groups showed significantly lower weight gain than those of the control group. (Fig. 2) The volume of water required to reach the AWR score of 3 (rat responded by lifting abdomen) in Groups C and D was significantly lower than that in the control group, indicating high visceral sensation in these two model groups.

**Fig. 3:**
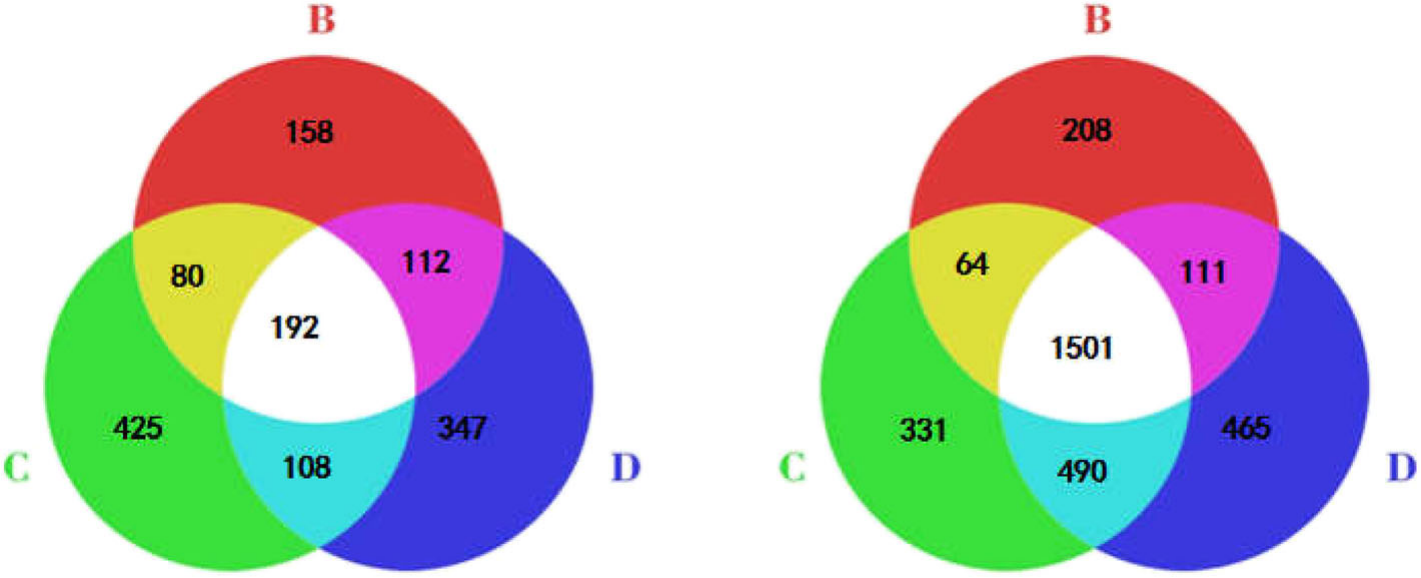
Venn diagram showing the differentially expressed proteins identified in the colon (left) and brain (right) of groups B, C and D

### Proteomic analysis

Using the labeled iTRAQ method, we performed global profiling of protein expression from the brain tissue and colon of three groups of rats. In total, 77,135 matched spectra resulted in 19,005 matched peptides assembled into 3064 proteins in the colon, and 98,087 matched spectra led to 23,081 matched peptides assembled into 3793 proteins in the brain. Differentially expressed proteins were defined by those with a > 1.2-fold difference in expression from the control group and *P* < 0.05, as shown in a Venn diagram in Fig. 3. The detailed information of differentially expressed proteins were shown in Additional files 2 and 3. In the colon, in comparison to group A (control), 542 differentially expressed proteins were identified in group B, among which 309 proteins were upregulated and 233 were downregulated; 809 differentially expressed proteins were identified in group C, among which 415 were upregulated and 394 were downregulated; group D exhibited 731 differentially expressed proteins, of which 424 were upregulated and 307 were downregulated; groups B and C presented more differentially expressed proteins than group A. There were significantly more differentially expressed proteins in the three model groups in the brain than in the colon. In the brain, 1884, 2386 and 2567 proteins were changed in groups B, C and D, respectively. Among the differentially expressed proteins, 764 proteins were upregulated and 1120 were downregulated in group B, 1080 were upregulated and 1306 proteins were downregulated in group C, and 1187 were upregulated and 1380 were downregulated in group D. Among groups B, C and D, the number of common differentially expressed proteins between the brain and colon were 153, 280, and 239, respectively. As shown in Table 1, in group B, among the common differentially expressed proteins in the colon and brain, 15 proteins were upregulated and 21 proteins were downregulated together; in group C, 44 proteins were upregulated and 96 were downregulated together; and in group D, 26 proteins were upregulated and 27 were downregulated together. Among the common differentially expressed proteins, groups B and C shared 17 proteins, groups B and D shared 38 proteins, groups C and D shared 47 proteins, and groups B, C and D shared 55 proteins (Table 1).

**Table 1:** Number of common differentially expressed proteins that were modified 2-fold (up- or downregulation) in different experimental groups

Expression location	Group		
	Group B	Group C	Group D
B,C↑	15	44	26
B,C↓	21	96	27
B↑C↓	36	60	70
B↓C↑	81	80	116
Total	153	280	239

a) *B* brain, *C* colon b) ↑:upregulated, ↓:downregulated

Furthermore, we inquired which of the differentially expressed proteins with the same expression change existed in both the brain and colon of the three IBS models and what their possible functions could be. As shown in Table 2, 43 differentially expressed proteins showed the same expression change in the three IBS models, including 25 proteins upregulated in the colon and downregulated in the brain (termed CU&BD), 7 proteins downregulated in the colon and upregulated in the brain (termed CD&BU), and 3 proteins upregulated in the colon and brain (termed CBU) and 8 downregulated in the colon and brain (termed CBD). In the biological process category, the proteins were found to participate in RNA binding, protein transport, lipid binding, the inflammatory response, the electron transport chain, DNA binding, cation binding, ATP binding, RNA binding, and calcium ion binding.

**Table 2:**
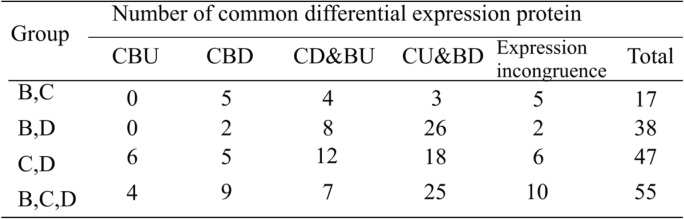
Number of common differential expression protein in two groups and three groups

a) CBU: upregulated in the colon and brain b) CBD: downregulated in the colon and brain. c) CU&BD: upregulated in the colon and downregulated in the brain d) CD&BU: downregulated in the colon and upregulated in the brain

### Pathway analysis

**Fig. 4:**
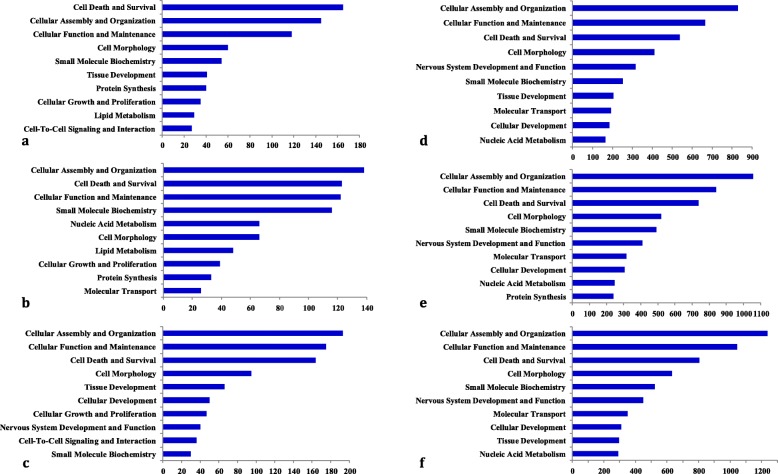
GO term distribution of the enriched proteins that were differentially expressed in the colon (**a**, **b**, **c**) and brain (**d**, **e**, **f**) of group A (**a**, **d**), B (**b**, **e**) and C(**c**, **f**). The stripes indicate the expected protein number for each functional group

According to GO, the differentially expressed proteins for each group were functionally annotated. Figure 4 and Table 3 display the significant GO terms, ranked by their significance level. Identified proteins based on iTRAQ labeling were divided into 46, 52, and 48 functional categories in the colon and 52, 51 and 55 functional categories in the brain for groups B, C and D, respectively. Most of the identified proteins were found to be involved in cellular assembly and organization, cellular function and maintenance and cell death and survival (Additional file 4).

**Table 3:** The information of the common differential expressed proteins from iTRAQ data and MS validation and the possible biological function for those three groups

Expression change	Protein name	Accession no.	Function /Biological_process
C↑B↓	Heterogeneous nuclear ribonucleoprotein H2	Q6AY09	RNA binding; nucleotide binding
C↑B↓	T-kininogen 2	P08932	cysteine-type endopeptidase inhibitor activity
C↑B↓	Protein Zyx	D4A7U1	metal ion binding;zinc ion binding
C↑B↓	Adaptin ear-binding coat-associated protein 2	Q6P756	/ protein transport
C↑B↓	Protein Ttc1	Q66H09	unknown
C↑B↓	NSFL1 cofactor p47	O35987	lipid binding; ubiquitin binding
C↑B↓	Toll-interacting protein	A2RUW1	/inflammatory response; innate immune response; signal transduction
C↑B↓	Cystatin-C	P14841	beta-amyloid binding; cysteine-type endopeptidase inhibitor activity; protease binding
C↑B↓	Microtubule-associated protein	F1LQB5	unknown
C↑B↓	Anamorsin	Q5XID1	/ apoptotic process
C↑B↓	Zero beta-globin (Fragment)	Q63011	heme binding; iron ion binding; oxygen binding; oxygen transporter activity
C↑B↓	LSM4 homolog, U6 small nuclear RNA associated (*S. cerevisiae*) (Predicted), isoform CRA_a NADH dehydrogenase	D4A2C6	unknown
C↑B↓	(Ubiquinone) flavoprotein 3-like, isoform CRA_a	G3 V644	unknown
C↑B↓	Microtubule-associated protein 1A	P34926	actin binding
C↑B↓	Ndufa7 protein	A9UMV9	NADH dehydrogenase (ubiquinone) activity
C↑B↓	PDZ and LIM domain protein 4	P36202	metal ion binding; zinc ion binding
C↑B↓	Atp8b2 protein	Q4V8A7	unknown
C↑B↓	UPF0449 protein C19orf25 homolog	Q6AY72	unknown
C↑B↓	Biphenyl hydrolase-like (Serine hydrolase)	Q3B8N9	hydrolase activity
C↑B↓	Ubiquitin-fold modifier 1 (Fragment)	G5C7K5	unknown
C↑B↓	NADH dehydrogenase [ubiquinone] flavoprotein 3, mitochondrial	Q6PCU8	/electron transport chain
C↑B↓	Purkinje cell protein 4 (Fragment)	G5BG09	unknown
C↑B↓	Ubiquitin-conjugating enzyme E2 B (Fragment)	G5BN13	ATP binding;acid-amino acid ligase activity
C↑B↓	Neuromodulin	P07936	/glial cell differentiation; nervous system development
C↑B↓	Protein Chchd2	E9PT03	unknown
C↓B↑	Histone H3.1	Q6LED0	DNA binding
C↓B↑	Galactosylceramidase	Q5YKG1	cation binding; galactosylceramidase activity
C↓B↑	Interferon-induced, double-stranded RNA-activated protein kinase	Q63184	ATP binding;double-stranded RNA binding;non-membrane spanning protein tyrosine kinase activity; protein serine/threonine kinase activity
C↓B↑	Histone H1.4	P15865	DNA binding
C↓B↑	Tyrosine--tRNA ligase, mitochondrial	Q5I0L3	ATP binding RNA binding tyrosine binding tyrosine-tRNA ligase activity
C↓B↑	Pre-mRNA processing factor 8, isoform CRA_a	G3V6H2	/mRNA splicing, via spliceosome
C↓B↑	Histone H3	D3ZK97	DNA binding
CB↑	Bifunctional epoxide hydrolase 2	P80299	10-hydroxy-9-(phosphonooxy) octadecanoate phosphatase activity; 4-nitrophenylphosphatase activity; epoxide hydrolase activity; magnesium ion binding
CB↑	60S ribosomal protein L23	P62832	structural constituent of ribosome
CB↑	NLR family member X1	Q5FVQ8	ATP binding
CB↓	Tropomyosin alpha-4 chain	P09495	metal ion binding
CB↓	Calmodulin	G5BS71	calcium ion binding
CB↓	Tropomyosin alpha-3 chain	Q63610	/ brain development
CB↓	Polypyrimidine tract-binding protein 1 (Fragment)	G5C5X1	RNA binding;nucleotide binding
CB↓	Cytochrome b-c1 complex subunit 6, mitochondrial	Q5M9I5	protein complex binding; ubiquinol-cytochrome-c reductase activity
CB↓	Glyceraldehyde 3-phosphate dehydrogenase (Fragment)	P97617	nucleotide binding;oxidoreductase activity; acting on the aldehyde or oxo group of donors; NAD or NADP as acceptor
CB↓	Protein Srrm1	D3ZD33	/ mRNA processing
CB↓	V-type proton ATPase subunit F	P50408	ATPase activity; hydrogen ion transporting ATP synthase activity; proton-transporting ATPase activity

a) *B* brain, *C* colon b) ↑:upregulated, ↓:downregulated

Based on functional annotation with GO, as shown in Fig. 4, the differentially expressed proteins in the colon and brain of the three IBS models displayed similar enrichment distributions in the functions of cellular assembly and organization and cellular function and maintenance.

**Fig. 5:**
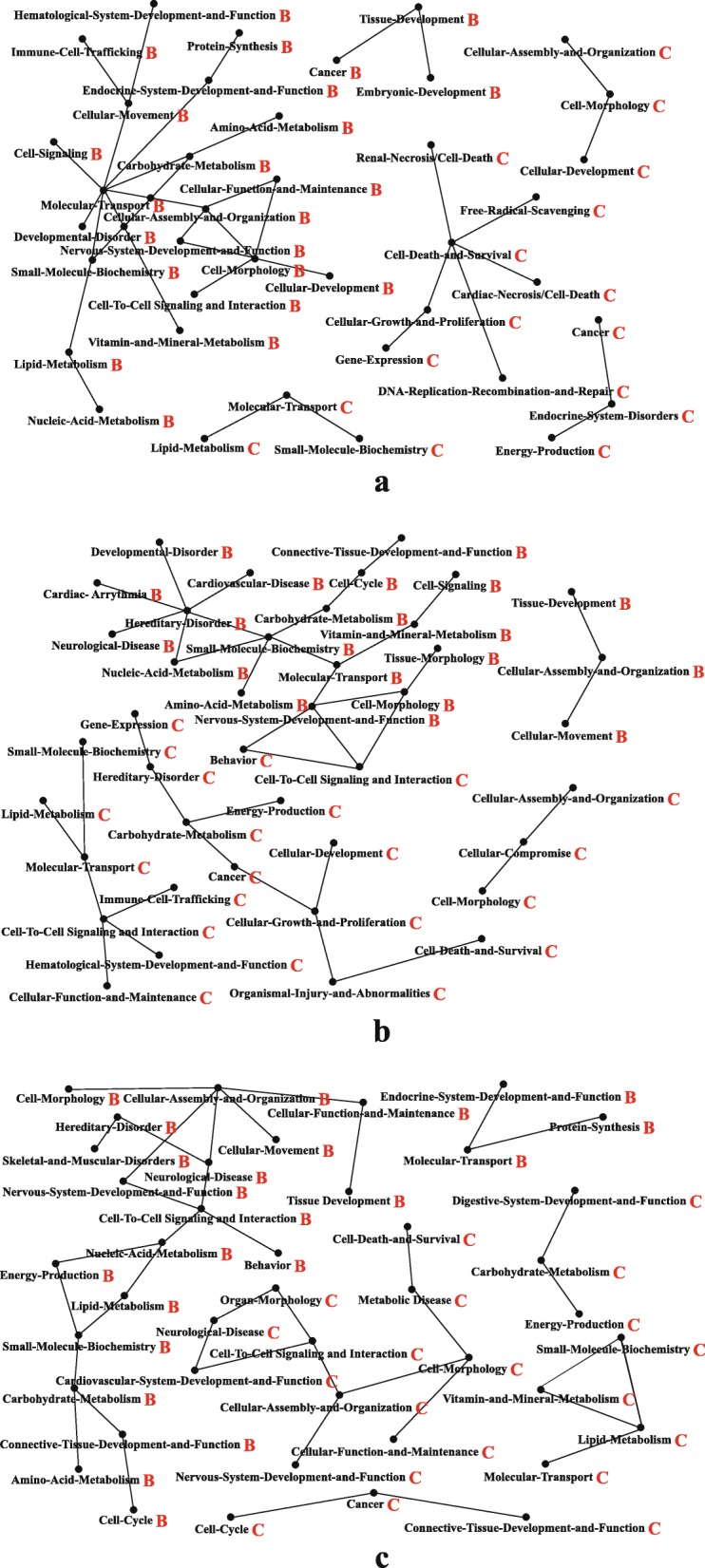
Protein-protein interaction networks in groups B (**a**), C (**b**) and D (**c**). B: brain, C: colon

As Table 4 and Table 5 show, the common signaling pathways from the colon in the three IBS models were granzyme A signaling, interleukin (IL)-4 signaling, mitochondrial dysfunction, the protein ubiquitination pathway and the superpathway of geranylgeranyl diphosphate biosynthesis. The common signaling pathways from the brain in the three IBS models could be grouped into 13 principal pathways, namely, 14–3-3-mediated signaling, breast cancer regulation by stathmin 1, clathrin-mediated endocytosis signaling, aldosterone signaling in epithelial cells, epithelial adherens junction signaling, glycolysis I, integrin signaling, mitochondrial dysfunction, modeling of epithelial adherens junctions, protein ubiquitination pathway, Sertoli cell-Sertoli cell junction signaling, synaptic long-term potentiation and TCA cycle II. The protein ubiquitination pathway and mitochondrial dysfunction were signaling pathways common to both the colon and the brain of the three IBS models. A recent study demonstrated reduced expression of colonic ubiquitinated proteins in IBS-D [22], confirming that the abnormal ubiquitination pathway plays a key role in the pathogenesis of IBS. By exploring the global possible protein-protein interactions (PPIs) (Fig. 5), we identified a group of GO terms including molecular transport, small molecular biochemistry, cell-to-cell signaling and interaction, and cellular assembly and organization enriched in the brains of the three IBS models. Compared to that in the brain, cell death and survival, cell-to-cell signaling and interaction and cell morphology in the colon were more involved in the three IBS models (Fig. 4).

**Table 4:** Common pathway in the colon of three groups

Pathway	Group
Granzyme A Signaling	BCD
IL-4 Signaling	BCD
Mitochondrial dysfunction	BCD
Protein Ubiquitination Pathway	BCD
Superpathway of Gernylgeranyldiphosphate Biosynthesis I (via Mevalonate)	BCD

**Table 5:** Common pathway in the brain of three groups

Pathway	Group
14–3-3-mediated Signaling	BCD
Breast Cancer Regulation by Stathmin 1	BCD
Clathrin-mediated Endocytosis Signaling	BCD
adosterone Signaling in Epithelial Cells	BCD
Epithelia Adherens Junction Signling	BCD
Glycolysis I	BCD
Integrin Signaling	BCD
Mitochondrial Dysfuncion	BCD
modeling of Epithelial Adherens Junctions	BCD
Protein Ubiquitination Pathway	BCD
Sertoli Cell-Sertoli Cell Junction Signaling	BCD
Synaptic Long Term Potentiation	BCD
TCA Cycle II (Eukaryotic)	BCD

### Confirmation of the differential proteins by Western blotting

**Fig. 6:**
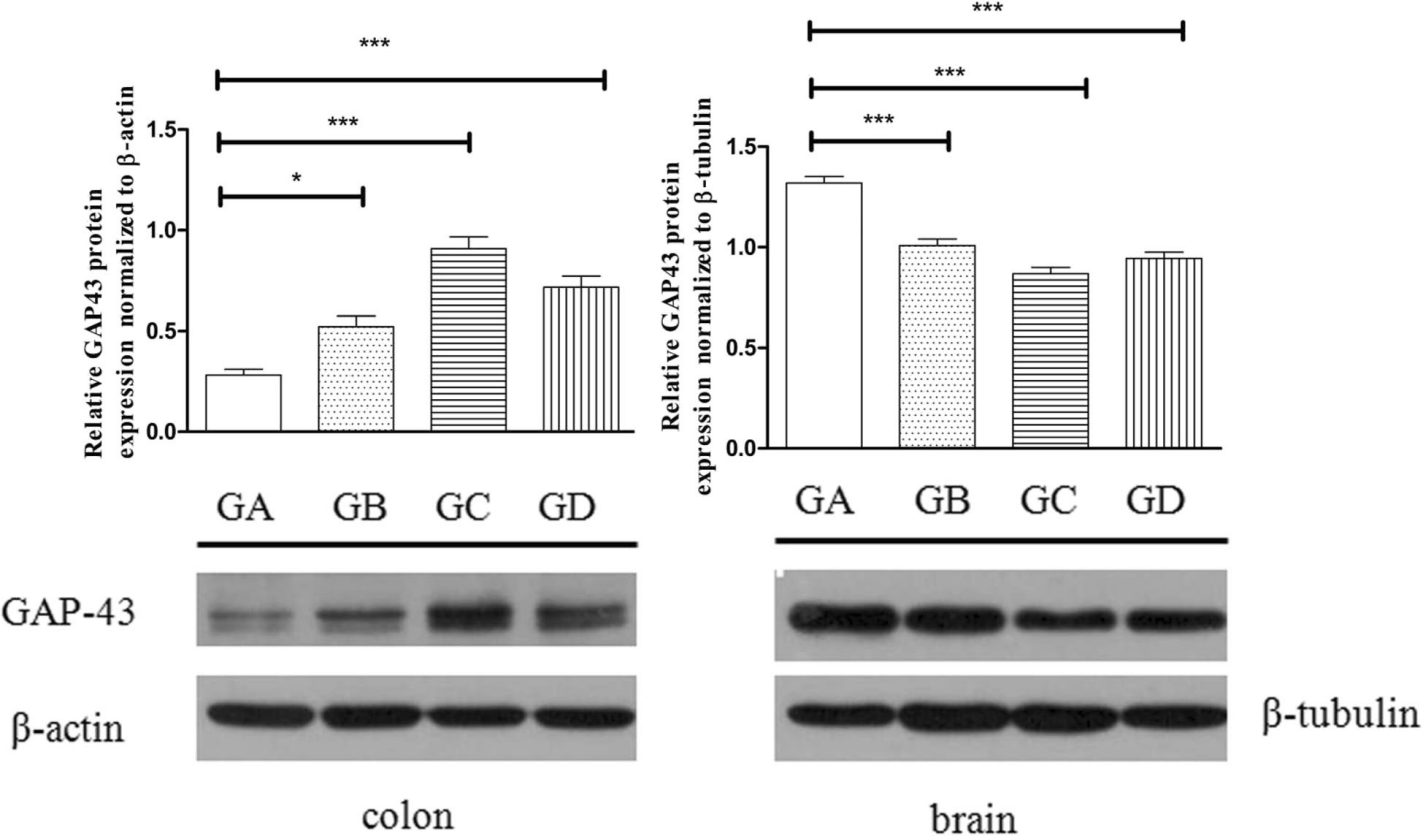
Western blotting detection of GAP-43 in the colon (left) and brain (right) of group A-D. Data are expressed as the mean ± SEM (*n* = 4). *P < 0.05 compared with normal control. (Group A, GA). ***P < 0.001 compared with normal control. (Group A, GA).GA: group A, GB: group B, GC: group C

Western blotting was performed to verify the expression of targeted proteins identified by the iTRAQ analysis. We selected the differentially expressed protein neuromodulin GAP-43 because it is considered to be associated with visceral hypersensitivity in IBS patients [23], and the results were consistent with the iTRAQ data (Fig. 6). These results demonstrate the satisfactory quality of our experimental procedures and data.

## Discussion

This study analyzed the differentially expressed protein profiles between the brain and colon in three IBS models. There have been some reports on differentially expressed proteins in the colon of IBS models [14, 15], similar to our study, and the present study contributes to this growing body of literature. However, we also detected differentially expressed proteins in the brain of IBS models. These results are new evidence of abnormal interactions in the BGA. The present study demonstrated that there are 153, 280 and 239 common differentially expressed proteins in the brain and colon of groups B, C and D, respectively. Overall, 35 common differentially expressed proteins exhibited > 2-fold expression change compared to that of the control group, and 23 of those proteins have a known function.

In the present study, we focused on the common differentially expressed proteins in both the brain and colon tissue of three IBS models, which might reflect a series of key factors involved in the physiopathological mechanism of visceral hypersensitivity. Three proteins were identified to be upregulated in the two tissues, including bifunctional epoxide hydrolase 2, 60S ribosomal protein L23 and the nucleotide binding domain and leucine-rich repeat-containing (NLR) family member X1 (NLRX1). NLRX1 has been shown to be an important regulator of critical pathways associated with both inflammation and tumorigenesis [24]. Recent reports have shown that NLRX1 plays an important role in neuronal apoptosis by increasing mitochondrial fission [25]. In the three IBS models, NLRX1 expression in both tissue types was higher than that in the control group. This difference likely results in CNS and ENS neuron abnormalities that might be involved in the physiopathological mechanisms of IBS.

Eight proteins were identified to be downregulated in the two tissue types, including tropomyosin alpha-4 chain, tropomyosin alpha-3 chain, calmodulin, polypyrimidine tract-binding protein 1, cytochrome bc1 complex subunit 6, glyceraldehyde 3-phosphate dehydrogenase, protein Srrm1, and V-type proton ATPase subunit F. In muscle, tropomyosin alpha-4 chain and tropomyosin alpha-3 chain play a central role in the calcium-dependent regulation of vertebrate striated muscle contraction. In nonmuscle, these proteins are involved in stabilizing cytoskeleton actin filaments. Calmodulin (CaM) mediates the control of a large number of enzymes, ion channels, aquaporins and other proteins by Ca^2+^.Calmodulin, an important molecule in Ca^2+^-CaM-calcium/calmodulin-dependent protein kinase II (CaMKII) signaling, plays an important role in chronic visceral pain [26, 27]. In the brain and colon of the three IBS models, calmodulin expression was decreased, showing that Ca^2+^-CaM-CaMKII might be a key pathway involved in the physiopathological mechanism of IBS. There were 7 proteins that were downregulated in the colon and upregulated in the brain, most of which are binding proteins. There were 25 proteins upregulated in the colon and downregulated in the brain. According to the predicted function of the 25 proteins, 8 proteins are binding proteins that function in RNA, metal ion, lipid, beta-amyloid, heme, actin, and ATP binding. The function of 9 proteins is unknown, and the other 8 proteins are involved in cysteine-type endopeptidase inhibitor activity, protein transport, the inflammatory response, the apoptotic process, nicotinamide adenine dinucleotide (NADH) dehydrogenase, hydrolase activity, the electron transport chain, and glial cell differentiation. Neuromodulin, a calmodulin-binding polypeptide, has been demonstrated to be beneficial to neuronal plasticity in the CNS [28–30]. Previous studies have demonstrated that GAP-43, a neuromodulin, is involved in the pathophysiology of depression and the mechanisms of antidepressants [31, 32]. Our study demonstrated that neuromodulin was decreased in the brains of the three IBS models with characteristics of brain-gut deregulation and showed that neuromodulin in the brain may play an important role in the pathogenesis of IBS. A recent study found that GAP-43, which is involved in visceral hypersensitivity, was increased in the mucosa of IBS patients [23], consistent with our study.

The present study has some limitations. In the future, the functions of the key differentially expressed proteins in both the brain and colon in IBS models should be investigated further. Next, we will attempt to examine the important factors identified in these brain-gut disorder animal models in IBS patients.

## Conclusions

Taken together, the data presented here represent a comprehensive and quantitative proteomic analysis of the brain and colon in IBS models, thereby deepening our understanding and providing new evidence of an abnormal brain-gut interaction in IBS.

## Supplementary information

**Additional file 1.** Peptide information of brain and colon for iTRAQ experiment.

**Additional file 2: Table S1.** Detailed information of differentially expressed proteins in colon of three groups.

**Additional file 3: Table S2.** Detailed information of differentially expressed proteins in brain of three groups.

**Additional file 4: Table S3.** Functional categories of identified protein in colon and brain of three IBS models.

## Data Availability

The datasets used and analyzed during the current study are available from the corresponding author upon reasonable request.

## Abbreviations

AWR: Abdominal withdrawal reflex
BGA: Brain-gut axis
CaM: Calmodulin
CaMKII: Calcium/calmodulin-dependent protein kinase II
CBD: Downregulated in the colon and brain
CBU: Upregulated in the colon and brain
CD&BU: Downregulated in the colon and upregulated in the brain
CU&BD: Upregulated in the colon and downregulated in the brain
CWRS: Chronic wrap restraint stress
ENS: Enteric nervous system
GAP-43: Growth-associated protein 43
GBA: Gut-brain axis
GI: Gastrointestinal
HPA: Hypothalamic-pituitary-adrenal
IBS: Irritable bowel syndrome
MS: Maternal separation
NADH: Nicotinamide adenine dinucleotide
NLR: Nucleotide binding domain and leucine-rich repeat-containing
PND: Postnatal day
