# Supplementary material for: Comparative proteomic analysis of the brain and colon in three rat models of irritable bowel syndrome
Source: Proteome Sci. 2020 Feb 24;18:1. doi: 10.1186/s12953-020-0157-9 (PMC7041085; doi:10.1186/s12953-020-0157-9)
Supplement: Supplementary file 4 — Additional file 4: Table S3. Functional categories of identified protein in colon and brain of three IBS models. [file 12953_2020_157_MOESM4_ESM.docx]

Results of functional categories in colon and brain of three IBS models

| functional categories | Colon | | | Brain | | |
| --- | --- | --- | --- | --- | --- | --- |
|  | (number of differentially expressed protein) | | | | | |
|  | GB | GC | GD | GB | GC | GD |
| Cellular Assembly and Organization | 145 | 138 | 193 | 829 | 1055 | 1237 |
| Cellular Function and Maintenance | 118 | 122 | 175 | 665 | 840 | 1043 |
| Cell Death and Survival | 165 | 123 | 164 | 538 | 737 | 805 |
| Small Molecule Biochemistry | 54 | 116 | 30 | 251 | 491 | 522 |
| Cell Morphology | 60 | 66 | 95 | 410 | 519 | 631 |
| Tissue Development | 41 | 10 | 66 | 205 | 233 | 297 |
| Nucleic Acid Metabolism | 7 | 66 | 4 | 165 | 247 | 291 |
| Cellular Development | 21 | 7 | 50 | 184 | 306 | 310 |
| Lipid Metabolism | 29 | 48 | 23 | 60 | 190 | 175 |
| Cellular Growth and Proliferation | 35 | 39 | 47 | 17 | 55 | 67 |
| Protein Synthesis | 40 | 33 | 21 | 154 | 241 | 253 |
| Nervous System Development and Function | 11 | 20 | 36 | 316 | 409 | 448 |
| Cell-To-Cell Signaling and Interaction | 27 | 9 | 36 | 91 | 126 | 139 |
| Molecular Transport | 13 | 26 | 17 | 193 | 315 | 349 |
| Cell Cycle | 10 | 8 | 23 | 11 | 17 | 54 |
| Skeletal and Muscular System Development and Function | 21 | 5 | 13 | 8 | 8 | 17 |
| Carbohydrate Metabolism | 20 | 17 | 6 | 45 | 94 | 89 |
| Amino Acid Metabolism | 0 | 20 | 2 | 20 | 65 | 60 |
| Organismal Development | 17 | 3 | 10 | 9 | 9 | 16 |
| Embryonic Development | 14 | 2 | 9 | 53 | 57 | 72 |
| Organ Development | 13 | 1 | 6 | 9 | 9 | 16 |
| Hematological System Development and Function | 9 | 4 | 12 | 0 | 0 | 5 |
| Energy Production | 8 | 11 | 1 | 27 | 67 | 77 |
| Cellular Compromise | 10 | 7 | 6 | 23 | 0 | 44 |
| Immune Cell Trafficking | 9 | 3 | 4 | 0 | 0 | 5 |
| DNA Replication, Recombination, and Repair | 3 | 9 | 3 | 7 | 14 | 20 |
| Protein Trafficking | 0 | 8 | 0 | 27 | 31 | 39 |
| Neurological Disease | 1 | 8 | 0 | 11 | 25 | 25 |
| Inflammatory Response | 8 | 3 | 6 | 0 | 2 | 7 |
| Endocrine System Disorders | 0 | 7 | 8 | 16 | 18 | 20 |
| Metabolic Disease | 0 | 7 | 7 | 16 | 18 | 20 |
| Gastrointestinal Disease | 0 | 7 | 7 | 16 | 18 | 20 |
| Cancer | 0 | 0 | 7 | 51 | 48 | 70 |
| Renal and Urological System Development and Function | 6 | 4 | 1 | 2 | 0 | 0 |
| Free Radical Scavenging | 6 | 0 | 1 | 51 | 49 | 71 |
| Cellular Movement | 6 | 3 | 6 | 41 | 93 | 136 |
| Cardiovascular System Development and Function | 6 | 6 | 5 | 4 | 2 | 4 |
| Post-Translational Modification | 4 | 5 | 2 | 21 | 59 | 109 |
| Endocrine System Development and Function | 1 | 1 | 5 | 0 | 0 | 0 |
| Reproductive System Development and Function | 4 | 3 | 4 | 0 | 0 | 0 |
| Organismal Injury and Abnormalities | 3 | 4 | 0 | 0 | 8 | 10 |
| Hair and Skin Development and Function | 3 | 2 | 4 | 5 | 14 | 19 |
| Drug Metabolism | 2 | 0 | 4 | 19 | 24 | 28 |
| Connective Tissue Development and Function | 4 | 4 | 3 | 3 | 23 | 29 |
| Vitamin and Mineral Metabolism | 2 | 1 | 3 | 5 | 15 | 2 |
| Tissue Morphology | 1 | 2 | 3 | 4 | 2 | 2 |
| Organismal Functions | 0 | 0 | 3 | 5 | 0 |  |
| Renal and Urological Disease | 0 | 0 | 2 | 0 | 5 | 8 |
| Infectious Disease | 0 | 2 | 0 | 0 | 0 | 0 |
| Hypersensitivity Response | 2 | 0 | 0 | 0 | 0 | 0 |
| Cell Signaling | 0 | 2 | 0 | 11 | 10 | 18 |
| Cardiovascular Disease | 2 | 1 | 1 | 0 | 6 | 9 |
| Antimicrobial Response | 0 | 0 | 2 | 0 | 0 | 0 |
| Visual System Development and Function | 0 | 1 | 0 | 0 | 0 | 0 |
| Organ Morphology | 1 | 1 | 0 | 4 | 2 | 4 |
| Ophthalmic Disease | 0 | 1 | 0 | 0 | 0 | 0 |
| Inflammatory Disease | 0 | 1 | 0 | 0 | 0 | 0 |
| Gene Expression | 1 | 1 | 0 | 0 | 0 | 0 |
| Digestive System Development and Function | 0 | 1 | 1 | 5 | 0 | 6 |
| Behavior | 1 | 0 | 0 | 11 | 12 | 12 |
| Antigen Presentation | 1 | 0 | 0 | 0 | 0 | 0 |
| Developmental Disorder | 0 | 0 | 0 | 8 | 8 | 11 |
| Immunological Disease | 0 | 0 | 0 | 2 | 0 | 0 |
| Protein Degradation | 0 | 0 | 0 | 21 | 38 | 24 |
| Protein Folding | 0 | 0 | 0 | 7 | 10 | 12 |
| RNA Damage and Repair | 0 | 0 | 0 | 2 | 2 | 2 |
| RNA Post-Transcriptional Modification | 0 | 0 | 0 | 2 | 2 | 2 |
| Skeletal and Muscular Disorders | 0 | 0 | 0 | 2 | 6 | 9 |

GA:group A GB: group B GC: group C
